# Socioeconomic position links circulatory microbiota differences with biological age

**DOI:** 10.1038/s41598-021-92042-0

**Published:** 2021-06-16

**Authors:** Hannah Craven, Dagmara McGuinness, Sarah Buchanan, Norman Galbraith, David H. McGuinness, Brian Jones, Emilie Combet, Denise Mafra, Peter Bergman, Anne Ellaway, Peter Stenvinkel, Umer Z. Ijaz, Paul G. Shiels

**Affiliations:** 1 Wolfson Wohl Translational Research Centre, Institute of Cancer Sciences, MVLS, Garscube Estate, University of Glasgow, Switchback Road, Glasgow, G61 1QH UK; 2 NHS GG&C, Glasgow, UK; 3 School of Medicine, University of Glasgow, Glasgow, UK; 4 Fluminense Federal University (UFF), Niterói, RJ Brazil; 5 Division of Renal Medicine M99, Department of Clinical Science, Intervention and Technology, Karolinska Institutet, Stockholm, Sweden; 6 Institute of Health and Wellbeing, MVLS, University of Glasgow, Glasgow, UK; 7 School of Engineering, University of Glasgow, Glasgow, UK; 8 Glasgow Polyomics, University of Glasgow, Glasgow, UK; 9 Institute of Infection, Immunity and Inflammation, University of Glasgow, Glasgow, UK

**Keywords:** Epidemiology, Microbiome

## Abstract

Imbalanced nutrition is associated with accelerated ageing, possibly mediated by microbiota. An analysis of the circulatory microbiota obtained from the leukocytes of participants in the MRC Twenty-07 general population cohort was performed. We now report that in this cohort, the most biologically aged exhibit a significantly higher abundance of circulatory pathogenic bacteria, including *Neisseria, Rothia* and *Porphyromonas,* while those less biologically aged possess more circulatory salutogenic (defined as being supportive of human health and wellbeing) bacteria, including *Lactobacillus*, *Lachnospiraceae UCG-004* and *Kocuria*. The presence of these salutogenic bactreria is consistent with a capacity to metabolise and produce Nrf2 agonists. We also demonstrate that associated one carbon metabolism, notably betaine levels, did not vary with chronological age, but displayed a difference with socioeconomic position (SEP). Those at lower SEP possessed significantly lower betaine levels indicative of a poorer diet and poorer health span and consistent with reduced global DNA methylation levels in this group. Our data suggest a clear route to improving age related health and resilience based on dietary modulation of the microbiota.

## Introduction

Accumulating evidence has indicated that exposome factors (i.e. psycho-social, dietary and lifestyle) significantly affect health span in the general population^1–4^. The mechanistic basis of this is complex and not well understood, though evidence indicates that diet and socioeconomic position (SEP) are major contributory factors^1,5–7^. An outstanding problem, however, has been to identify factors driving ‘‘inflammageing’’**.** which is not fully explained by an inflammatory burden attributable to the senescence associated secretory phenotype (SASP)^8–12^. As human microbiotal composition shows age related changes^4,8^, the microbial metabolite trimethylamine N-oxide (TMAO) has been proposed to affect both ‘inflammageing’ and health span^1,9–12^. Evidence in support of this hypothesis comes from observations of the frail elderly, where dietary differences were associated with differences in inflammatory burden and microbiotal composition^13^. This hypothesis, however, is complicated by the fact that TMAO is secreted in the urine and knowledge of renal function is therefore required to gauge true levels.

There are also further emerging roles for the microbiota in maintenance of the epigenetic landscape via the capacity of the gut microbiota to generate betaine from nutritional sources, thus providing a source of methyl donor groups that contribute to the maintenance of the methylome^14^.

We have previously demonstrated that accelerated ageing was exacerbated by low SEP and imbalanced diet in the general population^5–7^. Based on these observations, we have explored the hypothesis that imbalanced diet can drive changes in the microbiota that can adversely affect age-related health^7,15,16^.

As no faecal samples are available for retrospective analyses of these cohorts, including the MRC Twenty-07 cohort (the general population cohort used in this study, which is described in full here^17^), we have employed an analysis of the venous circulatory microbiome as a ‘*canary in the coal mine’* for changes in the gut microbiome, corresponding to extremes of biological age within the cohort. While the existence of a circulatory microbiome continues to be disputed, studies evidencing a blood microbiome have been accumulating in recent years, supporting the thesis that microbial signatures can and do exist in the blood, outwith any source of infection^18–22^. Bacterial ingress into the blood circulation is an inherent feature of gut leakiness, which increases with both age and as a feature of diminishing renal function^23–25^. It provides a snapshot of what is in the gut and what may impact on health span. This snapshot approach using the circulatory microbiome has recently been valorised and validated in an investigation of a range of cancers^15^, and rheumatoid arthritis (RA)^26^. We have therefore sought to correlate features of such a putative blood microbial complement with metadata from the MRC Twenty-07 cohort, in order to determine if there are differences in the microbiota correlated with SEP, inflammation and the landscape of ageing in the general population.

## Materials and methods

### Cohort details

Data were from the West of Scotland Twenty-07 Study, a community-based, prospective cohort study, which has followed three cohorts of men and women recruited in 1987 at the (approximate) ages of 35 (‘1970s cohort’), 55 (‘1950s cohort’) and 75 years (‘1930s cohort’) in 1987 (wave 1) and followed up in a further four waves over the next 20 years until 2007/8. Details of this cohort, the study design, its biochemical, biophysical and socioeconomic characterization have been described in depth elsewhere^17^. The Tayside Committee on Medical Research Ethics approved the study, and all research involving the use of human data was performed in accordance with relevant guidelines/regulations. Informed, written consent was obtained from all subjects (all over 18 years old) at each wave of the study. Data and blood samples (final wave only) were collected by trained nurses in the homes of the study participants, when respondents were aged approximately 35 (1970s cohort), 55 (1950s cohort) and 75 (1930s cohort) years. Data from this study are available upon request. A measure of area deprivation (Carstairs deprivation score), an index based on census variables on area levels of overcrowding, no car access, male unemployment and low social class^27^ was used to assign SEP. Telomere analysis of this cohort has been described in depth elsewhere^3,17,28^. A sub-sample of n = 100 cases were selected from this MRC Twenty-07 general population cohort for this study. Samples (2 × n = 50) represented the extremes of biological age and SEP; i.e. individuals at low SEP displaying the shortest 50 telomere lengths in the MRC Twenty-07 cohort, compared with those at high SEP displaying the longest sample telomere lengths.

### Quantification of elements of one carbon metabolism

Plasma heparin samples were obtained after a 12 h fast and stored at -80 °C. Quantification of TMAO, choline and betaine was performed by LC–MS/MS as done by Missailidis^29^, utilizing a protocol designed specifically for this purpose and prepared in a 96-well format*.* Extracted plasma aliquots were spiked with internal standards, comprised of TMAO-D9 in methanol and water with Proline-13C5 as a recovery standard, and injected on an Agilent 1290 Infinity chromatographic system (Agilent Technologies, Waldbronn, Germany) fitted with an Acquity UPLC Amide column in combination with a VanGuard precolumn (Waters Corporation, Milford, MA, USA). The compounds were detected with an Agilent 6490 Triple Quadrupole mass spectrometer (Agilent Techologies, Santa Clara, CA, USA). Data processing was performed with MassHunter Quantitative Analysis QQQ (Agilent Technologies Inc. Santa Clara, CA, USA). The MS/MS analyses for TMAO, choline, betaine, TMAO- D9 and Proline-13C5, were conducted in multiple-reaction-monitoring (MRM) mode at *m/z* 76 → 58, *m/z* 104 → 45, *m/z* 118 → 58, *m/z* 85 → 66 and *m/z* 121 → 74 respectively.

### DNA isolation and 16S amplicon library preparation for microbiome analysis

DNA was extracted from peripheral blood leukocytes using Maxwell®16 System (Maxwell® 16 Blood DNA Purification kit, Promega), and quantified using the High Sensitivity DNA Qubit system (ThermoFisher, Paisley, UK). 16S libraries encompassing the V3-V4 regions were generated by Glasgow Polyomics as done in Taponen^30^. Briefly, the V3 to V4 regions of bacterial 16S were amplified using Kapa HiFi Hotstart Readymix (2 ×) (Kapa Biosystems, Wilmington, MA, USA) with the addition of primers specific for the V3 and V4 regions of 16S (based on the standard Illumina 16S primers), which contain an overlap sequence making the primers compatible with the Nextera XT indexing reagents (Illumina, San Diego, CA, USA). Samples were then amplified using a 5 min 95 °C hotstart followed by 26 cycles of 95 °C for 30 s and 60 °C for 1 min with a final elongation step of 60 °C for 5 min. The resulting amplicons were purified using bead extraction (SPRI select beads, Beckman Coulter, Brea, CA, USA), using 0.9 × beads followed by 80% ethanol washes and resuspension in 10 mM Tris–EDTA buffer. The amplicons were quantified using the High Sensitivity DNA Qubit system and profiles were obtained from an Agilent 2100 Bioanalyser using High Sensitivity DNA reagents (Agilent, Santa Clara, CA, USA). Samples were then standardized to 10 ng per reaction and amplified in the presence of Nextera XT v2 indexes using Kapa HiFi Hotstart readymix (2 ×) for 8 cycles. The resulting indexed libraries were then purified and quality controlled as before. The libraries were combined in equimolar ratios and sequenced on a MiSeq (Illumina, San Diego, CA, USA) instrument using a paired end, 2 × 300 bp, sequencing run. Samples were sequenced with an average of 50 000 reads per sample. Possible contamination of reagents was controlled for by running a negative control sample (Nuclease-Free water (Ambion™, AM9932, Thermo Fisher Scientific)), instead of a DNA sample through the whole analysis, in conjunction with the true test samples. Water only samples were treated identically to test samples. Five sample libraries were not deemed suitable for sequencing due to the extremely low DNA concentrations, leading to a total of 95 out of the original 100 DNA libraries being successfully sequenced.

### Bioinformatics

#### Sequence quality trimming and OTU generation

Bioinformatics was carried out as in Ijaz^31^. Paired-end reads were trimmed and filtered using Sickle v1.200 using a sliding window approach, trimming the reads where the average base quality dropped below 20. Only reads that followed the default quality criteria after trimming in Sickle were kept, followed by error-correction of the paired-end reads by BayesHammer from V2.5.0 assembler. Following this, pandaseq (2.4) was used to assemble the forward and reverse reads into a single sequence spanning the entire V3/V4 region, with a minimum overlap of 10 bp. These preprocessed reads (overlapped) from each sample were pooled together, with barcodes added for identification. The reads were then de-replicated, sorted in order of decreasing abundance and singletons were discarded. Next, the VSEARCH v2.3.4 software was used to generate the abundance table by constructing Operational Taxonomic Units (OTUs) by clustering of the reads based on 97% similarity, followed by a two-step chimera removal stage using the(–uchime_denovo option in vsearch), and a reference based chimera filtering step using a gold database (https://www.mothur.org/w/images/f/f1/Silva.gold.bacteria.zip). Finally, OTU tables for each sample were generated by matching the original barcoded reads against clean OTUs at 97% similarity (a proxy for species-level separation). Having obtained the OTUs, DeConseq was used to identify OTUs that were contaminants, hitting on human reference genome^32^.

The assign_taxonomy.py script from the Qiime workflow^33^ was used to taxonomically classify the representative OTUs against the SILVA SSU Ref NR database release v123 database. Following this, the OTUs were multisequence aligned using MAFFT v 7.3^34^ and were used in FastTree v2.1.7^35^ to generate the phylogenetic tree in NEWICK format. The biom file for the OTUs was then generated by combining the abundance table with taxonomy information using make_otu_table.py from the Qiime workflow.

### Statistical analyses

Statistical analyses were performed in R as by Ijaz^31^. The vegan package was used for alpha and beta diversity analyses. For alpha diversity measures we have used: Shannon entropy—a commonly used index to measure balance within a community, and rarefied richness (exponential of Shannon entropy)—the estimated number of species. Ordination of OTU table in reduced space (beta diversity) was done using Principal Coordinate Analysis (PCoA) plots of OTUs using three different distance measures: Bray–Curtis, Unweighted Unifrac and Weighted Unifrac (shown). Unifrac distances were calculated using the phyloseq package^36^. Analysis of variance for explanatory variables (or sources of variation) was performed using Vegan’s adonis() against distance matrices (Bray–Curtis/UnweightedUniFrac/Weighted UniFrac). This function, referred to as PERMANOVA, fits linear models to distance matrices and used a permutation test with pseudo-F ratios to explain variability in the microbial community structure, contingent upon the variation in the given extrinsic parameter of interest.

To find OTUs that were significantly different between multiple categories considered in this study, we used the DESeq2 package^37^ with the adjusted *p* value significance cut-off of 0.05 and log fold change cut-off of 2.0. This function uses negative binomial GLM fitting to obtain maximum likelihood estimates for the OTUs log fold change between the two conditions. Bayesian shrinkage was then applied to obtain shrunken log fold changes, subsequently employing the Wald test for obtaining significances.

Correlation analysis between one carbon metabolism elements and the rest of the metadata^38^ was performed using the Kendall rank correlation coefficient, and Bonferonni adjustment was used to generate the p values. R’s fdrtool^39^ was also used as an alternative adjustment method (seen in supplementary materials Fig. 1). The scripts and workflows used to carry out all of the above bioinformatics and analyses can be found at http://userweb.eng.gla.ac.uk/umer.ijaz#bioinformatics.

## Results

### TMAO and choline increase with chronological age

TMAO was measured in 100 samples from the MRC Twenty-07 general population cohort, representing the extremes of biological age and SEP; i.e. individuals at low SEP displaying the shortest 50 telomere lengths (n = 50), compared with those at high SEP displaying the longest sample telomere lengths (n = 50). Sample ages covered the range 35–75 years. Of the patients included in this analysis, only two were on diabetes medication.

**Figure 1:**
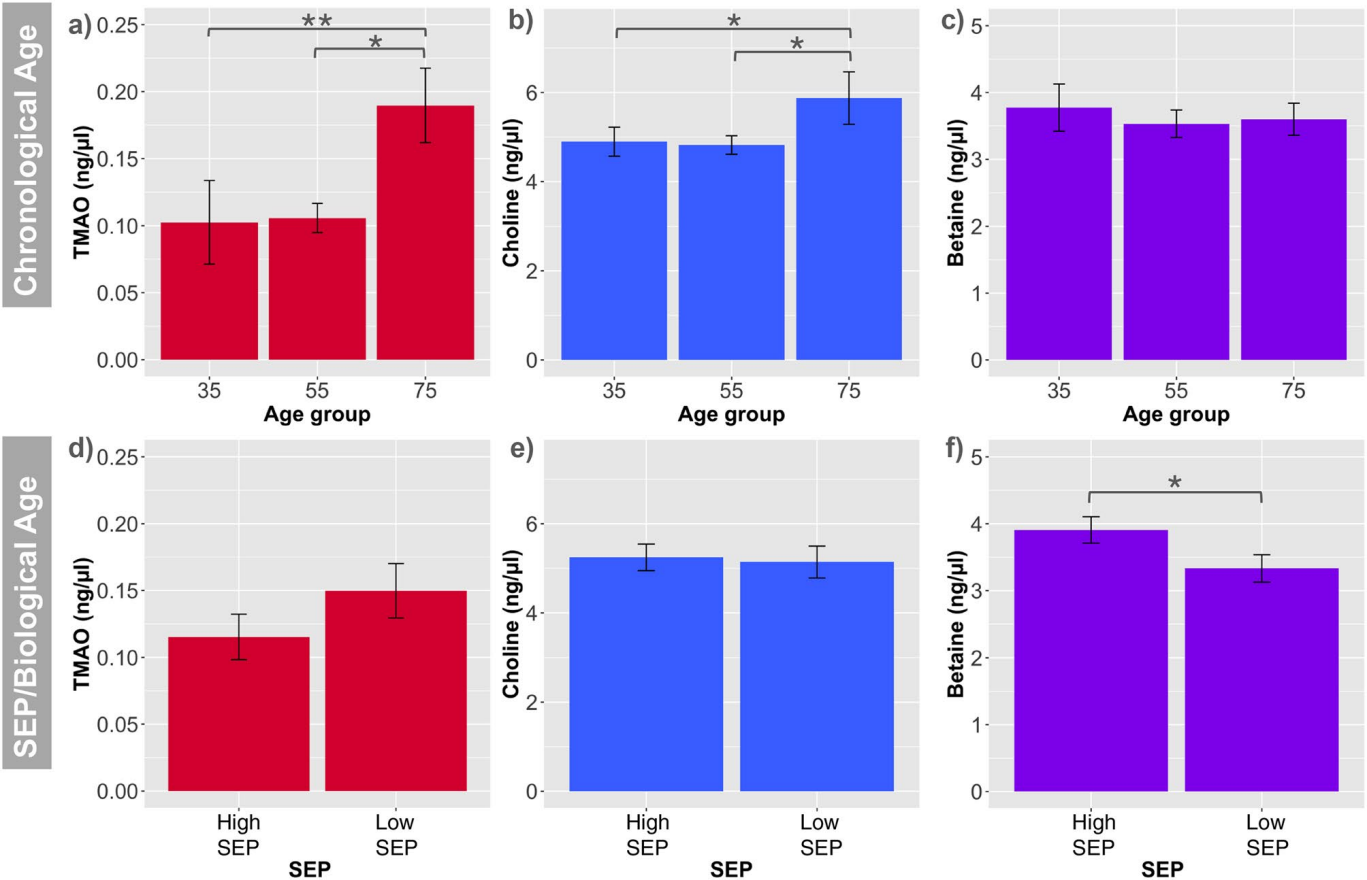
Changes in elements of one carbon metabolism with chronological and biological age/SEP. Variations in mean TMAO, betaine and choline levels across 3 tertiles of chronological age (**a**–**c**). Variations of TMAO, Betaine and Choline levels across two extremes of biological age and SEP, grouped by those that are the least deprived with the longest telomeres (High SEP) and the most deprived with the shortest telomeres (Low SEP) (**d**–**f**). Error bars represent standard error of the mean. ANOVA and TukeyHSD tests were performed to generate adjusted p values for multiple comparisons. Where significance occurred between groups, graphs were labeled as follows: *p* < 0.05 = *, *p* < 0.01 = **.

In this general population cohort TMAO and choline levels did not correlate with SEP (Fig. 1). They did, however, show a significant correlation with chronological age. TMAO values in serum increased significantly only between chronological age groups and not biological age (i.e. telomere length)/SEP groups even when adjusted for eGFR (Fig. 1a/d). No significant differences were observed between the low and high SEP groups for TMAO levels (Fig. 1d). However, there was a significantly higher TMAO level in the oldest-age group (~ 75 years old) compared to both the middle-age (~ 55 years old) and the lowest-age (~ 35 years old) groups, with *p* values < 0.05 and 0.01, respectively (Fig. 1a). Analogous to the TMAO results, no detectable differences in serum choline levels were observed between the low and high SEP groups (Fig. 1b). However, again there was a significantly higher choline level in the oldest-age group compared to the two younger groups (*p* < 0.05 for both). There were no significant differences found in betaine levels between any of the chronological age groups (Fig. 1c). In contrast, we have demonstrated a SEP difference for betaine levels, whereby those at low SEP had lower betaine levels in comparison to those at high SEP (*p* < 0.05) (Fig. 1f).

### One-carbon metabolism: correlations with MRC Twenty-07 metadata

Investigations to determine any associations between TMAO, betaine and choline levels and biophysical, bio-social and biochemical variables in the MRC Twenty-07 cohort, indicated that TMAO only showed a statistically significant correlation with chronological age (Table 1). No significant correlations were observed with any other variables, as can be seen in the full metadata correlations analysis in Supplementary materials—Fig. 1. Betaine showed significant positive correlation with choline level. Choline displayed significant positive correlations with betaine levels, mean corpuscular volume (MCV), and creatinine. A negative correlation was also observed between choline and estimated glomerular filtration rate (eGFR) (Table 1).

**Table 1:** Significant correlations between MRC Twenty-07 meta-variables and elements of one carbon metabolism.

One-Carbon metabolism element	Significant variable	Kendall correlation	Adjusted *p* value
TMAO	Age	0.32	0.0014**
Choline	Estimated Glomerular Filtration Rate (eGFR)	− 0.28	0.0142*
	Betaine	0.35	0.0002***
	Mean corpuscular volume (MCV)	0.28	0.0145*
	Creatinine	0.33	0.0007***
Betaine	Choline	0.35	0.0002***

Bonferroni adjustment was used to generate adjusted *p* values, where *** =  < 0.001, ** =  < 0.01, * =  < 0.05.

### Differential abundance of salutogenic bacteria correlates with SEP

**Figure 2:**
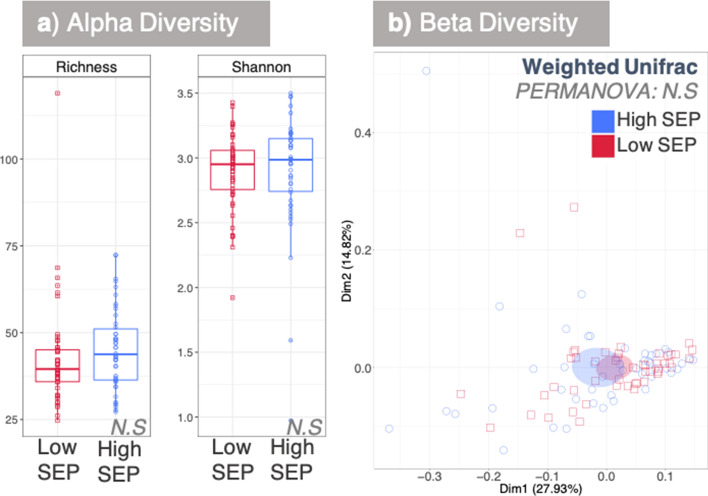
Microbial diversity analysis between high and low SEP. (**a**) and (**b**) represent alpha diversity and beta diversity indices, respectively. In (**b**), the ellipses are drawn at 95% confidence interval of standard error.

**Figure 3:**
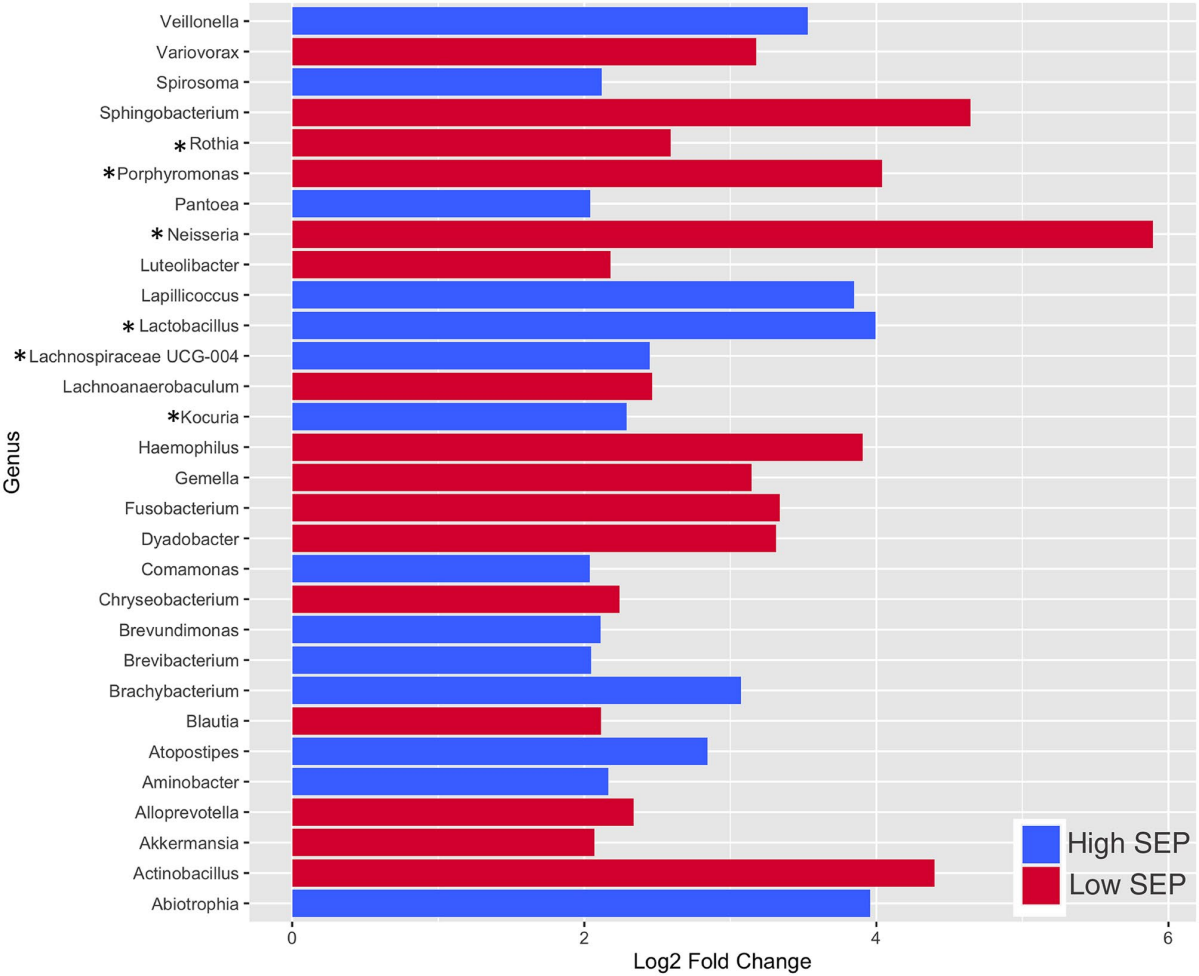
Differentiated Taxa Analysis showing Log2 changes in discriminant genera between the High and Low SEP groups, where * represents genera identified as being of particular interest. All discriminant genera shown have an adjusted *p* value < 0.001.

Of the 100 samples from the MRCTwenty-07 cohort that were selected for one-carbon metabolism analysis, 95 were successfully sequenced. No significant difference was found between the overall diversities (alpha and beta) of the microbial communities in the respective low and high SEP groups (Fig. 2a,b). However, 30 discriminant genera were identified through differential taxa analysis (Fig. 3), including some ‘salutogenic’ bacteria (i.e. beneficial to good health). Such bacteria include *Lactobacillus, Lachnospiraceae UCG-004* and *Kocuria,* which were all present at higher relative abundance in the high SEP group. Genera increased in the low SEP group included pathogenicity related *Neisseria, Rothia* and *Porphyromonas.*

### PERMANOVA analysis: vegetable intake, glycated haemoglobin and kidney function are sources of variation for the microbiota; TMAO and red meat are not

PERMANOVA analysis (Table 2) of the metadata associated with this cohort identified several significant sources of variation for the overall microbial diversity of the samples (measured using three metrices: Bray–Curtis, Weighted Unifrac and Unweighted Unifrac). The full list of metadata used in this analysis, including those that were not significant is shown in Supplementary materials Table 1.

**Table 2:** PERMANOVA analysis showing significant sources of variation (metadata) in microbial community structure (distances between samples) with R^2^ representing percentage variability if significant for that variable.

Variable	Bray–Curtis	Weighted Unifrac	Unweighted Unifrac
eGFR	R^2^ = 0.04359 (*p* = 0.0277) (*)	R^2^ = 0.04827 (*p* = 0.0644) (.)	n.s
Phosphate	R^2^ = 0.01658 (*p* = 0.0616) (.)	R^2^ = 0.02581 (*p* = 0.0328) (*)	R^2^ = 0.01554 (*p* = 0.0566) (.)
Choline	R^2^ = 0.02582 (*p* = 0.0022) (**)	R^2^ = 0.04775 (*p* = 0.0024) (**)	R^2^ = 0.01623 (*p* = 0.0479) (*)
Betaine	n.s	R^2^ = 0.02235 (*p* = 0.0451) (*)	n.s
Haemoglobin	n.s	R^2^ = 0.02022 (*p* = 0.061) (1)	n.s
Red blood cells	R^2^ = 0.0157 (*p* = 0.0691) (.)	n.s	n.s
Haematocrit	R^2^ = 0.01675 (*p* = 0.0464) (*)	R^2^ = 0.02768 (*p* = 0.0137) (*)	n.s
Lymphocytes	n.s	R^2^ = 0.01946 (*p* = 0.0702) (.)	n.s
Monocytes	R^2^ = 0.01655 (*p* = 0.0545) (.)	n.s	n.s
Eosinophils	n.s	R^2^ = 0.02429 (*p* = 0.0334) (*)	R^2^ = 0.02173 (*p* = 0.002) (**)
Creatinine	R^2^ = 0.01557 (*p* = 0.072) (.)	R^2^ = 0.01878 (*p* = 0.0861) (.)	R^2^ = 0.01434 (*p* = 0.0827) (.)
Hba1c	R^2^ = 0.01932 (*p* = 0.0372) (*)	R^2^ = 0.04314 (*p* = 0.0099) (**)	R^2^ = 0.01605 (*p* = 0.0449) (*)
Globulin	R^2^ = 0.01716 (*p* = 0.0572) (.)	n.s	n.s
Triglycerides	n.s	R^2^ = 0.02388 (*p* = 0.0526) (.)	n.s
Carstairs 2001 score	R^2^ = 0.01673 (*p* = 0.0367) (*)	n.s	n.s
Depcat 2001 score	R^2^ = 0.01709 (*p* = 0.0304) (*)	n.s	n.s
Head of household occupational social class	R^2^ = 0.01539 (*p* = 0.0696) (.)	R^2^ = 0.01902 (*p* = 0.0743) (.)	n.s
Vegetable frequency	R^2^ = 0.01487 (*p* = 0.0873) (.)	R^2^ = 0.02084 (*p* = 0.0559) (.)	n.s

For example, R^2^ = 0.02581 for phosphate implies 2.5% variability. Asterisks denote a statistically significant difference (*p < 0.05, **p < 0.01, ***p < 0.001). *n.s* denotes a non-statistically significant outcome (*p* > 0.05) and outcomes where the p value is a trend it is marked with an (.) where the *p* value is between 0.05 and 0.1.

Microbial diversity was found to associate with two measures of social deprivation (DEPCAT and Carstairs), albeit only when using one of the three diversity matrices employed in the analysis (Bray–Curtis *p* = 0.03). With PERMANOVA, significant association was identified between the microbiota and choline (Weighted Unifrac-*p* = 0.0024) and betaine levels (*p* = 0.0451), whilst no significant association was observed for TMAO. Significantly, the only food component showing a trend with microbiome diversity was vegetable intake (*p* = 0.0559), whereas neither fruit, nor red meat consumption showed any association (Supplementary materials-Table 1). differences in diversity of microbiota within this cohort correlated with several biochemical features, such as with glycated haemogobin (HbA1c), which proved to have a significant association with all 3 measures of beta diversity (e.g. Weighted Unifrac-*p* = 0.0099). A number of renal-related parameters showed association with variation in the microbiota of the the MRC Twenty-07 cohort, including eGFR (Bray Curtis-*p* = 0.0277), S-creatinine (Bray Curtis-*p* Trend = 0.072), and phosphate (Weighted Unifrac-*p* = 0.0328). Various blood cell types also showed a trend to association with microbial diversity, including eosinophils which strongly associated with beta diversity when measured by Weighted Unifrac (*p* = 0.0334) and Unweighted Unifrac (*p* = 0.002).

After categorising the cohort based on frequency of red meat and fish consumption (as sources of TMAO substrates), we measured beta diversity differences in the circulatory microbiota between high, medium and low intake groups (Supplementary Fig. 2a,e). PERMANOVA revealed no significant differences between any group for either food source. We also compared mean TMAO, choline and betaine levels between these groups, to determine if red meat and white fish consumption had any effect on elements of one carbon metabolism (Supplementary Fig. 2b–d,f–h). While TMAO did increase in both the high red meat and high fish consumption groups, ANOVA did not confirm significance for this comparison, or any other.

## Discussion

We have demonstrated in a general population cohort that TMAO and choline levels do not correlate with SEP. They did, however, show a significant correlation with chronological age. This was not influenced by SEP, the composition of the microbiota, or biological age as measured by telomere length. In contrast, we have demonstrated a SEP difference for betaine levels. This is in keeping with published reports of an accelerated biological ageing and diminished global methylation content among those at low SEP in an overlapping demographic^6^. Notably, our metadata analysis also showed that betaine levels contribute to the small variations in the microbiota of individuals of varying SEP (Table 2). Higher betaine plasma levels are associated with high intake of betaine-rich food sources, such as quinoa, spinach, fortified cereal products, wheat germ, bran, and beets, as well as with synthesis from choline oxidation^14^. As intake of fruit and vegetables are lower in low SEP groups^40^, this may partly explain the low betaine plasma levels. As more salutogenic foods are more expensive^41^, low income associated with lower SEP has been associated with intake of foods characterized by a high-energy low-nutrient content. Conversely, high SEP has been associated with more health-conscious food intake^5,42^ and the consumption of so-called ‘*superfoods*’ as a marketed vehicle for added nutrition (e.g. spelt, quinoa and goji berries, chia seeds or wheatgrass)^43^.

The lack of association between SEP, the microbiota and TMAO levels is not intuitive, given recent literature indicating that TMAO is a feature of inflammageing and disease^1,12,29^. Our data suggests that lack of adjustment of chronological and/or, biological age, may be a confounder for these analyses. Furthermore, our data are from a general population cohort and not one tied to a specific morbidity and thus more reflective of normative ageing. This is supported by our microbiota analyses, which have indicated that those at differing SEP and biological age within the same demographic, do not show differences in overall microbial diversity (Fig. 2a,b). None of the 30 discriminant genera identified between the high and low SEP groups (Fig. 3) are known TMAO-producers^44^, strengthening the finding that SEP does not influence TMAO levels.

Our differential taxa analysis (Fig. 3) indicates that there is a greater prevalence of *Lactobacillus* among those at higher SEP and low biological age. This genus has the capacity to process (poly)phenolic acids derived from the dietary intake of fruit and vegetables, to generate alkyl catechols^45^. Alky catechols are potent Nrf2 agonists and therefore have the capacity to regulate the expression of > 390 anti-oxidant genes^46^. Additionally, *Lactobacillus* has the capacity to derive betaine via nutritional precursors^47^ influencing the maintenance of the epigenome (methylome). These findings are thus consistent with better physiological robustness and health span among those at higher SEP as a consequence.

Our analysis indicated that *Lachnospiraceae UCG-0*04 are more prevalent in the high SEP group. Species from the *Lachnospiraceae* genus are known butyrate-producers abundant in the colon^48^. Being the primary energy source for colonocytes, butyrate plays a key role in maintaining gut epithelial integrity and is also another known activator of the NrF2 pathway^49^. In addition, the anti-carcinogenic effects of butyrate have been widely reported, as it is able to modulate gene expression via epigenetic regulation (e.g. histone deacetylation and DNA methylation) and impact upon apoptosis and cell cycle inhibitors such as p21 and BAK^50^. The prevalence of butyrate-producing bacteria in this group suggests that those of high SEP may benefit more from a microbial landscape that promotes butyrate-mediated protection against cancer, and other age-associated diseases induced by oxidative stress and a loss of epigenetic regulation.

*Kocuria* was also found to be more prevalent in the high SEP group. This genus is one of few bacteria identified as a potent producer of microbially-derived β-cryptoxanthin (β-CRX)^51^. This is a naturally occurring pro-vitamin A carotenoid found in only a limited number of fruit and vegetables such as oranges, papayas and sweet peppers^52^. The oral administration of this carotenoid has gained interest recently, due to widely reported health benefits, such as its anti-inflammatory, anti-obesity and anti-diabetic effects. Significantly, β-CRX has been shown to activate the Nrf2 and NfkB pathways^52^. Whilst *Kocuria* has been established as a β-CRX producer under optimal fermentation conditions in the lab, it is unclear whether this extant in the gut microbiota in vivo, and would require further exploration. However, the prevalence of an additional genus within the high SEP group that has the potential to produce a Nrf2 agonist is consistent with improved health span and physiological resilience in this group.

In addition to the “pro-health” genera observed in the high SEP cohort, our analyses also indicated an increase in pathogenic bacteria among the microbiota of the lower SEP group. These individuals showed increased numbers of bacteria associated with periodontal disease, such as *Rothia* and *Porphyromonas*^53,54^. Periodontitis has been linked to several systemic diseases within the diseasome of ageing, including chronic kidney disease, cardiovascular disease, cancer and type 2 diabetes (T2D)^55,56^. Furthermore, we observed an increase in the abundance of *Neisseria* in the low SEP group, consistent with poorer renal health. *Neisseria* is a genus that has previously been associated with CKD^57^. The dominance of these genera, along with the loss of any bacteria capable of producing agonists to anti-oxidative pathways indicates that those of poor SEP have a potential bias towards a circulatory microbiome that promotes poor physiological health.

PERMANOVA analysis (Table 2) of the metadata associated with this cohort identified several sources of variation, which impacted the overall microbial diversity of the samples. Significantly, microbial diversity was found to associate with two measures of deprivation (DEPCAT and Carstairs), albeit only when using one of the three diversity matrices employed in the analysis (Bray–Curtis). This indicates that whilst the deprivation status of an individual may not impact the phylogenetic variation of the associated microbiota, as indicated by both Unifrac measures (i.e. the distances between samples based on the branch lengths of the species present), it can impact upon the abundance of each species found.

The finding from the metadata analysis that frequency of red meat consumption did not show any associations with the microbiota or changes in beta diversity (Supplementary Fig. 2a) was surprising, given the literature on the impact of red meat on the gut microbiota and its metabolism^9,58,59^. There was also no change in beta diversity when categorised by white fish consumption (another dietary component associated with TMAO levels^60,61^), and whilst TMAO consumption did appear to increase in the highest white fish and red meat intake groups, these changes were not statistically significant (Supplementary Fig. 2). Instead, our analysis indicated that vegetable intake was a possible source of variation. Whilst we are conscious that our study has limited power, it is pertinent that we find biologically plausible associations between microbial composition and vegetable intake. The mechanistic basis of such an association may be derived from (poly)phenolic acids found in fruit and vegetables which are metabolised into smaller, more bioavailable molecules, including alkyl catechols, by some of the bacteria found to be more prevalent in the high SEP group, such as *Lactobacillus.* As this bacteria is a producer of Nrf2 agonists, activation of the anti-oxidative Nrf2 pathway may be promoted by a diet rich in fruit and vegetables^45^, which is more common in the high SEP group^62^. Furthermore, dietary carbohydrates that undergo bacterial fermentation into butyrate, another agonist of the Nrf2 pathway are also found in fruit and vegetables, and absent from meat^63^. Our findings provide a potential mechanistic link between the importance of plant foods in the diet, the microbiome, and the improved health span that is observed in those at high SEP. Interestingly, our analysis seems to suggest that vegetable consumption has more impact on the microbiota than red meat, as neither TMAO, nor red meat per se, associated with any changes in microbial diversity in this cohort. This analysis therefore provides no evidence that the burden of ‘inflammageing’ on health span and the age-related epigenome can be linked to microbially-derived TMAO in a general population cohort.

A recent study has evaluated SEP with composition of the gut microbiome in 1672 healthy volunteers from twin registry TwinsUK and observed that low SEP was associated with unhealthy diet and reduced alpha diversity measures, but diet did not explain the effects of low SEP on gut microbiota profile and socioeconomic position. Thus, SEP may be an important confounding factor in microbiota evaluation studies^64^.

The differences in diversity of microbiota within this cohort correlated with several pathological features, such as with HbA1c, a widely used biomarker for metabolic control in T2D^65^. A number of studies have already reported that microbial dysbiosis in the gut is a significant driver of insulin resistance in T2D, through microbial metabolites that modulate host metabolic signalling pathways^66^. In addition, oral supplementation with *Lactobacillus reuteri GMNL-263* has been reported to ameliorate the effects of insulin resistance in rats fed a high fructose diet, demonstrated by a decrease in multiple T2D markers, including HbA1c^67^. The finding that HbA1c is a factor associated with variation for the microbiota in this cohort, along with the finding that the *Lactobacillus* genus is enriched in the high SEP group (Fig. 3) suggests that those at high SEP may have a more favorable microbiome in terms of enabling optimal energy output from the diet, and protecting against insulin resistance.

Kidney function was also shown to be factor associated with variation in the microbiota of the MRC Twenty-07 cohort, in keeping with previous studies showing that microbial dysbiosis has a role in kidney disease progression^68^. A number of blood cell types also correlated with microbial diversity, including eosinophils. The association of these specific leukocytes with alterations to microbial diversity within this cohort, further supports the growing hypothesis that they may influence maintenance of endothelial barrier function and thus the gut microbial environment^69,70^.

In conclusion, our data indicate that being at lower SEP is not linked to microbial dysbiosis assessed by analysis of the circulatory microbiome, as we observed no difference in overall diversity compared to those of high SEP. This is not surprising, given that this is a general population cohort rather than one tied to any specific disease. Despite this, we have identified several differences in the discriminant genera prevalent within the two groups that are informative. Combined with our metadata analysis, we can propose a mechanistic link between the higher vegetable intake of those at higher SEP, and the provision of Nrf2 agonists due to the coding-capacity of *Lactobacillus* and *Kocuria* prevalent in this group. This should intuitively provide cyto-protection and promote better health span. Conversely, this protection is lost in the lower SEP group, with a lower relative intake of fruit and vegetables, and with an increased prevalence of bacteria that have the potential to contribute to systemic age-related diseases. However, it is important to note at this stage that this is merely a hypothesis worth investigating in future work. Additionally, a link between the high red meat intake of the low SEP, and microbially-produced TMAO could not be established as a contributing factor to the burden of ‘inflammageing’ in the general population, at least at current power of analysis, with vegetable intake having a seemingly higher impact on physiological health.

## Supplementary Information

Supplementary Information.
