# Supplementary material for: Socioeconomic position links circulatory microbiota differences with biological age
Source: Sci Rep. 2021 Jun 16;11:12629. doi: 10.1038/s41598-021-92042-0 (PMC8209159; doi:10.1038/s41598-021-92042-0)
Supplement: Supplementary file 1 — Supplementary Information. [file 41598_2021_92042_MOESM1_ESM.docx]

Socioeconomic position links circulatory microbiota differences with biological age

Hannah Craven^1§^, Dagmara McGuinness^1§^, Sarah Buchanan^1^, Norman Galbraith^2^, David H. McGuinness^8^, Brian Jones^2^, Emilie Combet^3^, Denise Mafra^4^ Peter Bergman^5^, Anne Ellaway^6^, Peter Stenvinkel^5^, Umer Z. Ijaz^7^ and Paul G. Shiels^1^

1. Institute of Cancer Sciences, MVLS, University of Glasgow, UK.

2. NHS GG&C, Glasgow, UK

3. School of Medicine University of Glasgow, UK

4.Fluminense Federal University (UFF), Niterói, RJ, Brazil

5. Division of Renal Medicine M99, Department of Clinical Science, Intervention

& Technology, Karolinska Institutet, Stockholm, Sweden

6. Institute of Health and Wellbeing, MVLS, University of Glasgow, UK

7. School of Engineering University of Glasgow, UK.

8. Glasgow Polyomics, University of Glasgow, UK

9. Institute of Infection, Immunity & Inflammation, University of Glasgow, UK

**Corresponding authors:**

**Biology:** Prof. Paul G Shiels

University of Glasgow, Wolfson Wohl Translational Research Centre, Institute of Cancer Sciences, Garscube Estate, Switchback Road, Glasgow, G61 1QH

E-mail: [paul.shiels@glasgow.ac.uk](mailto:paul.shiels@glasgow.ac.uk)

Computational biology: Umer Z. Ijaz , School of Engineering University of Glasgow, UK

E-mail: umer.ijaz@glasgow.ac.uk

§: These authors contributed equally to this work

**Classification**

BIOLOGICAL SCIENCES: Population biology

**Keywords**

Social Deprivation, Ageing, Microbiome, Nutrition, One carbon metabolism

**Author Contributions**

PGS conceived and designed the study, DMcG, DHMcG, SB, NG, PB, UZI and HC performed practical analyses. DM, BJ, AE, EC, PS, DMcG, HC and PGS discussed and interpreted the data. All authors contributed to manuscript writing.

**Fig 1.** The heatmaps shows the full correlation analysis between elements of One-carbon metabolism (TMAO, Choline and betaine) and metadata from the MRC20-07 cohort calculated using the Kendall rank correlation coefficient for a) dietary and social parameters, and b) biochemical parameters of interest. The p-values from the correlation analysis were adjusted for multiple comparisons (accounting for all comparison within a strip) using the traditional Bonferroni method

as well as through the recent R’s fdrtool^[[1]](#footnote-1)^♦. The significant positive (pink) or negative (blue) correlations are marked with * (*Adj. P* < 0.05), ** (*Adj. P* < 0.01) or *** (*Adj. P* < 0.001)


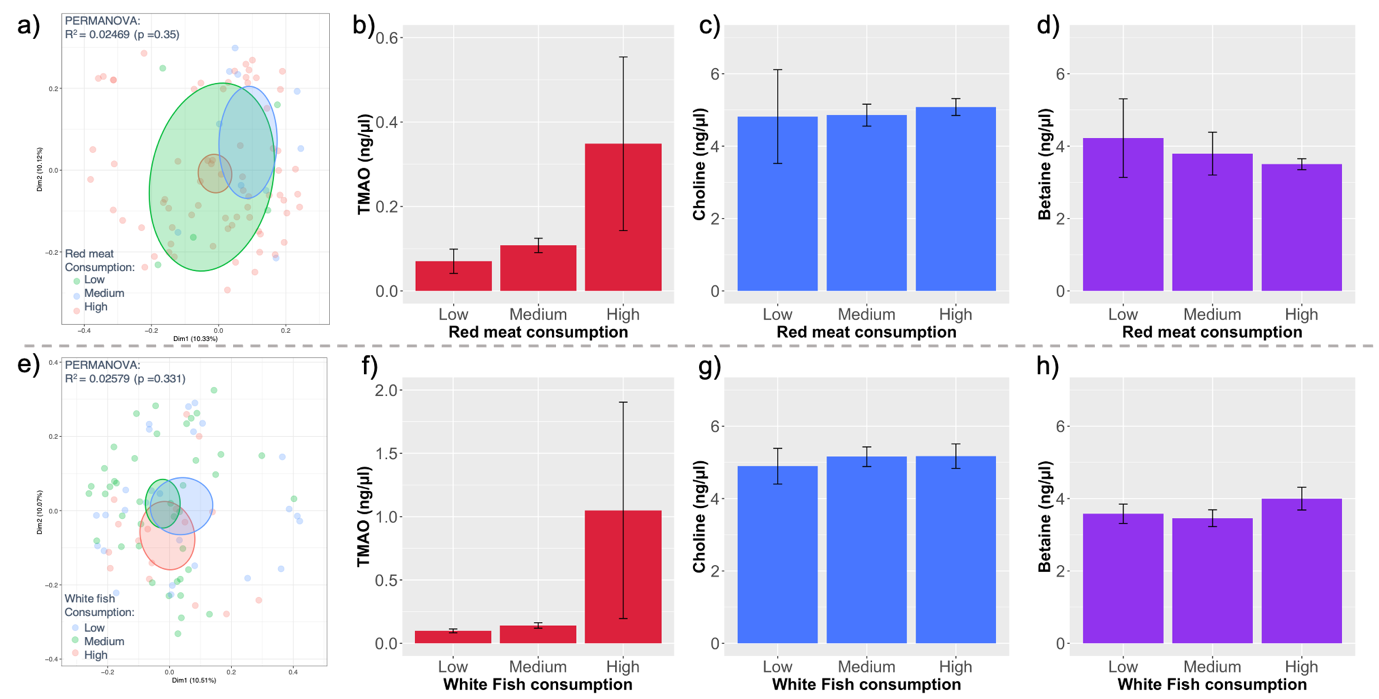


**Fig 2. Effect of red meat and fish consumption on circulatory microbiota diversity and one carbon metabolism.** (a) and (e) show beta diversity (Bray-Curtis metric) of the MRC Twenty-07 cohort when categorized by red meat and white fish consumption, respectively. In (b), the ellipses are drawn at 95% confidence interval of standard error. Error bars represent standard error of the mean. ANOVA and TukeyHSD tests were performed to generate adjusted p values for multiple comparisons. No p values generated between any of the groups were significant (p<0.05).

**Table 1.** PERMANOVA analysis showing sources of variation (metadata) in microbial community structure (distances between samples) with R^2^ representing percentage variability if significant for that variable. For example, R^2^=0.02581 for Phosphate implies 2.5% variability. Asterisks denote a statistically significant difference (*p < 0.05, **p < 0.01, ***p < 0.001). *n.s* denotes a non-statistically significant outcome (p>0.05) and outcomes where the p value is on the border of significance is marked with an (.) where the p value is between 0.05 and 0.1.

| **Variable** | **Bray-Curtis** | **Weighted Unifrac** | **Unweighted Unifrac** |
| --- | --- | --- | --- |
| eGFR | R^2^= 0.04359(p=0.0277)(*) | R^2^= 0.04827(p=0.0644)(.) | *n.s.* |
| Age | *n.s.* | *n.s.* | *n.s.* |
| TMAO | *n.s.* | *n.s.* | *n.s.* |
| Phosphate | R^2^=0.01658(p=0.0616)(.) | R^2^=0.02581(p=0.0328)(*) | R^2^=0.01554(p=0.0566)(.) |
| Telomere length | *n.s.* | *n.s.* | *n.s.* |
| Choline | R^2^=0.02582(p=0.0022)(**) | R^2^=0.04775(p=0.0024)(**) | R^2^=0.01623(p=0.0479)(*) |
| Betaine | *n.s.* | R^2^= 0.02235(p=0.0451)(*) | *n.s.* |
| SIMD quart | *n.s.* | *n.s.* | *n.s.* |
| White blood cells | *n.s.* | *n.s.* | *n.s.* |
| Haemoglobin | *n.s.* | R^2^=0.02022(p=0.061)(.) | *n.s.* |
| Red blood cells | R^2^= 0.0157 (p=0.0691)(.) | *n.s.* | *n.s.* |
| Haematocrit | R^2^=0.01675(p=0.0464)(*) | R^2^=0.02768(p=0.0137)(*) |  |
| Neutrophils | *n.s.* | *n.s.* | *n.s.* |
| Lymphocytes | *n.s.* | R^2^= 0.01946(p=0.0702)(.) | *n.s.* |
| Monocytes | R^2^= 0.01655(p=0.0545)(.) | *n.s.* | *n.s.* |
| Eosinophils | *n.s.* | R^2^=0.2429(p=0.0334)(*) | R^2^=0.2173(p=0.002)(**) |
| Basophils | *n.s.* | *n.s.* | *n.s.* |
| Fibrin | *n.s.* | *n.s.* | *n.s.* |
| C-reactive protein | *n.s.* | *n.s.* | *n.s.* |
| Urea | *n.s.* | *n.s.* | *n.s.* |
| Creatinine | R^2^=0.01557(p=0.072)(.) | R^2^=0.01878(p=0.0861)(.) | R^2^=0.01434(p=0.0827)(.) |
| Hba1c | R^2^=0.01932(p=0.0372)(*) | R^2^=0.04314(p=0.0099)(**) | R^2^=0.01605(p=0.0449)(*) |
| Leptin | *n.s.* | *n.s.* | *n.s.* |
| Bilirubin | *n.s.* | *n.s.* | *n.s.* |
| AST | *n.s.* | *n.s.* | *n.s.* |
| ALT | *n.s.* | *n.s.* | *n.s.* |
| GGT | *n.s.* | *n.s.* | *n.s.* |
| Protein | *n.s.* | *n.s.* | *n.s.* |
| Albumin | *n.s.* | *n.s.* | *n.s.* |
| Globulin | R^2^=0.01716(p=0.0572)(.) | *n.s.* | *n.s.* |
| Cholesterol | *n.s.* | *n.s.* | *n.s.* |
| Triglycerides | *n.s.* | R^2^=0.02388(p=0.0526)(.) | *n.s.* |
| HDL | *n.s.* | *n.s.* | *n.s.* |
| Red meat frequency | *n.s.* | *n.s.* | *n.s.* |
| Red meat product frequency | *n.s.* | *n.s.* | *n.s.* |
| Fast food in week frequency | *n.s.* | *n.s.* | *n.s.* |
| Fast food in past week | *n.s.* | *n.s.* | *n.s.* |
| Carstairs 2001 score | R^2^= 0.01673(p=0.0367)(*) | *n.s.* | *n.s.* |
| Depcat 2001 score | R^2^= 0.01709(p=0.0304)(*) | *n.s.* | *n.s.* |
| White fish frequency | *n.s.* | *n.s.* | *n.s.* |
| Sausages frequency | *n.s.* | *n.s.* | *n.s.* |
| Total alcohol units | *n.s.* | *n.s.* | *n.s.* |
| Alcohol units over 21 males | *n.s.* | *n.s.* | *n.s.* |
| Alcohol units over 14 females | *n.s.* | *n.s.* | *n.s.* |
| Employment status | *n.s.* | *n.s.* | *n.s.* |
| Head of household occupational social class | R^2^= 0.01539(p=0.0696)(.) | R^2^= 0.01902(p=0.0743)(.) | *n.s.* |
| Head of household source of income | *n.s.* | *n.s.* | *n.s.* |
| Vegetable frequency | R^2^= 0.01487(p=0.0873)(.) | R^2^= 0.02084(p=0.0559)(.) | *n.s.* |
| Fruit frequency | *n.s.* | *n.s.* | *n.s.* |
| Days without vegetables | *n.s.* | *n.s.* | *n.s.* |
| Fruit and Vegetable consumption | *n.s.* | *n.s.* | *n.s.* |
| SIMD score | *n.s.* | *n.s.* | *n.s.* |
| Aspirin | *n.s.* | *n.s.* | *n.s.* |
| Hormone replacement therapy | *n.s.* | *n.s.* | *n.s.* |
| Cortisol | *n.s.* | *n.s.* | *n.s.* |
| SSRI | *n.s.* | *n.s.* | *n.s.* |
| Statins | *n.s.* | *n.s.* | *n.s.* |
| Beta blockers | *n.s.* | *n.s.* | *n.s.* |
| Diuretics | *n.s.* | *n.s.* | *n.s.* |
| Diabetes medication | *n.s.* | *n.s.* | *n.s.* |
| Hypertensive medication | *n.s.* | *n.s.* | *n.s.* |
| BMI WHO class | *n.s.* | *n.s.* | *n.s.* |
| BMI WHO class 4 | *n.s.* | *n.s.* | *n.s.* |

Table 2. Definitions of abbreviations seen in MRC TWENTY-07 metadata

| **Abbreviation** | **Description/units measured** |
| --- | --- |
| Relative T.L | Relative telomere length |
| WBC_e | White blood Cell, x10^9/L (Full Blood Count (FBC) test) |
| HB_e | Haemoglobin, g/dL (FBC test) |
| RBC_e | Red blood cells, x10^12/L (FBC test) |
| HCT_e | Haemocitrit, l/L (FBC test) |
| MCV_e | Mean Corpuscular Volume, fl (FBC test). |
| MCH_e | Mean Corpuscular Haemoglobin, pg (FBC test). |
| RDW_e | red cell distribution width, % (FBC test). |
| PLT_e | Platelets, x10^9/L (FBC test) |
| Neut_e | Neutrophils, x10^9/L (FBC test) |
| Lymph_e | Lymphocytes, x10^9/L (FBC test) |
| Mono_e | Monocytes, x10^9/L (FBC test) |
| Eos_e | Eosinophiles, x10^9/L (FBC test) |
| Baso_e | Basophiles, x10^9/L (FBC test) |
| Fibrin_e | Fibrinogen, g/L |
| CRP_e | C-reactive protein, mg/L |
| Urea_e | Urea, mmol/L(kidney function test) |
| Creatinine_e | Creatinine, mmol/L(kidney function test) |
| HbA1c_e | Glycated haemoglobin, % |
| Leptin_e | Leptin, ng/mL |
| Bili_e | Bilirubin, µmol/L (liver function test) |
| APhos_e | Alkaline Phosphatase, U/L (liver function test) |
| AST_e | aspartate aminotransferase, U/L (liver function test). |
| ALT_e | alanine aminotransferase , U/L (liver function test). |
| GGT_e | *Gamma-* glutamyl transferase*,* U/L (liver function test). |
| Protein_e | Protein, g/L (liver function test) |
| Albumin_e | Albumin, g/L (liver function test) |
| Globulin_e | Globulin, g/L (liver function test) |
| Chol_e | Cholesterol, mmol/L (lipids) |
| Trig_e | Triglycerides, mmol/L (lipids) |
| HDL_e | HDL cholesterol, mmol/L (lipids) |
| Hohsc | Head of household occupational social class using RG1980 classification |
| Hohsource_e | Source from which 1980RG code was derived (n.b. male/female refers to respondents gender not gender of person from whom code derived) |
| Bmi_e | BMI continuous score |
| Obese_e | BMI 30 and over |
| Carstairs_2001_e | 2001 Carstairs continuous score. A measure of material deprivation within Scotland. The scores were constructed from four census variables: car ownership, male unemployment, overcrowding and low social class. |
| Depcat_2001_e | Depcat for wave 5 based on the 2001 census. A composite area-based indicator of socioeconomic status. It is derived from categories created from variables collected at the 2001 national census at postcode sector level comprising proportions of: residents living in overcrowded households; unemployed males; persons in households headed by someone of low social class; and, persons who do not own a car. |
| Dredm_e | If you do eat meat, how often do you usually have each kind? red meat (such as beef, lamb, pork (including bacon and ham)). Scale of 1(more than once a day) to 7(less than once a month or never). |
| Dmepro_e | If you do eat meat, how often do you usually have each kind? meat products (such as sausages, sausage rolls, meat pies, bridies, pasties, canned meat (e.g. corned beef) or burgers). Scale of 1(more than once a day) to 7(less than once a month or never). |
| Dtake_e | In the last 7 days how many times did your main meal of the day come from a takeaway or fast food seller of some kind? |
| Redmeatrecorded_e | red meat consumption wave 5 (1 = ">1 per day - > 1 per week", 2 = "less often or never" , 3 = "1-3 times per month") |
| Dwfisf_e | How often do you eat white fish (such as cod, haddock, whiting, sole or plaice, including fresh or frozen fish)? Scale of 1(more than once a day) to 7(less than once a month or never). |
| Dofisf_e | How often do you eat other types of fish (such as herring, mackerel, tuna, salmon or kippers, including fresh, frozen or canned fish)? Scale of 1(more than once a day) to 7(less than once a month or never). |
| Eats_sausages_e | eats sausages (1=at least several days per week, 2 = twice per month or less) |
| Alcohol_units_total_e | units of alcohol over previous week |
| Alc_unitsover21_males_e | alcohol units over 21 (males) |
| Alc_unitsover14_females_e | alcohol units over 14 (females) |

1. ♦ The fdrtool calculates both local and tail area-based false discovery rate (FDR) values for p-value statistics and fits empirical null models taking account of potential over- or under dispersion of the theoretical null distribution. The proportion of null values and the parameters of the null distribution are adaptively estimated from the data and therefore this adjustment method is recommended. [↑](#footnote-ref-1)
